# A hybrid technique for speech segregation and classification using a sophisticated deep neural network

**DOI:** 10.1371/journal.pone.0194151

**Published:** 2018-03-20

**Authors:** Khurram Ashfaq Qazi, Tabassam Nawaz, Zahid Mehmood, Muhammad Rashid, Hafiz Adnan Habib

**Affiliations:** 1 Department of Software Engineering, University of Engineering & Technology, Taxila, Pakistan; 2 Department of Computer Engineering, Umm Al-Qura University, Makkah, Saudi Arabia; 3 Department of Computer Science, University of Engineering & Technology, Taxila, Pakistan; University of South Carolina, UNITED STATES

## Abstract

Recent research on speech segregation and music fingerprinting has led to improvements in speech segregation and music identification algorithms. Speech and music segregation generally involves the identification of music followed by speech segregation. However, music segregation becomes a challenging task in the presence of noise. This paper proposes a novel method of speech segregation for unlabelled stationary noisy audio signals using the deep belief network (DBN) model. The proposed method successfully segregates a music signal from noisy audio streams. A recurrent neural network (RNN)-based hidden layer segregation model is applied to remove stationary noise. Dictionary-based fisher algorithms are employed for speech classification. The proposed method is tested on three datasets (TIMIT, MIR-1K, and MusicBrainz), and the results indicate the robustness of proposed method for speech segregation. The qualitative and quantitative analysis carried out on three datasets demonstrate the efficiency of the proposed method compared to the state-of-the-art speech segregation and classification-based methods.

## Introduction

The rapid growth of open-source multimedia content in the past few decades demands the development of efficient audio and visual content analysis techniques. Speech segregation and recognition from audio visual content, available either online and offline, depends on the quality and content of the audio signal [1]. Available audio content can contain noise; thus, musical segments can refer to the problem area during audio content analysis, especially in the case where speech segregation is needed. During the last decade, significant research solutions have been found but the challenge remains. Noise garbles speech and introduces obstacles in various applications, including automatic speech segregation. Noise removal from audio speech signals enhance the accuracy of speech recognition and segregation applications [2].

Existing methods of speech and music segregation use either learning-based methods or non-learning-based methods [1]. Learning-based methods have better classification accuracy when compared to non-learning-based methods; however, the accuracy comes at the expense of increased computational complexity. Learning-based methods are employed more frequently than non-learning-based methods because of their potential for segregating speech and music components more effectively in the presence of background noise. Lekshmil and Sathidevi [1] proposed non-learning-based speech segregation models for single-channel speech separation using short-time Fourier transform (STFT) [2]. They use pitch information-based techniques for the segregation process. Time-frequency mask-based pitch frequencies are gathered using dominant and interfering speaker information [2]. Cheng et al. and Hu et al. [3,4] proposed co-channel speech segregation using a non-learning-based model. They introduced a two-stage model segregation process. In the initial stage, a tandem algorithm is employed for simultaneous grouping. Then, a sequential grouping method for clustering is applied. Unvoiced speech is segregated first using onset and offset analysis. In the speech segregation step, binary masking is employed [5]. A two-stage model for ideal binary mask prediction was proposed by Kim et al. [6]; KNN was used for feature dimension prediction. When the output dimension was high, then the training of one DNN per output dimension is not scalable. In the proposed method, this problem is addressed by using the deep Boltzmann machine (DBM), where samples are trained over huge databases using multidimensional features. Websdale and Milner [7] proposed a method based on RNN. RNN is applied for speech segregation using the noisy audio sample. Speech segregation is performed using auditory masking. Samui et al. [5] introduced the critical band masking methods for the masking process. Earlier ideal binary masking (IBM) techniques are commonly used in auditory scene analysis (ASA) and computational ASA (CASA). GMM-based classification targeted unimpaired listeners, while DNN-based classification targeted impaired listeners. Wang et al. [8] proposed a multilayer perception-based classification method and trained algorithms using pitch-based features. Cho et al. [9] used GMM for the classification of amplitude modulation (AMS) features. They targeted dominant features and then classified time-frequency units via a Bayesian classification method. Pitch or harmonic structures are prominent features of voice speech segregation. Pitch-based features are very effective for IBM and voice segregation. For unvoiced/instrumental segregation, harmonic features are used.

The human auditory system segregates sound using a process known as auditory scene analysis (ASA) [10]. ASA analyses and recovers single and individual sound from a mixture of sounds to produce meaningful speech elements after removing noise elements. ASA is tough and complex because of the complex structure of the ear, which accesses only single pressure waves from different sources. The main functionality of ASA is that it segregates the elements of different sound sources and groups the elements from the same sound sources. In ASA, two steps are involved. The first one is segregation and the other is grouping [11]. In the first step, an input signal is decomposed into frames. Each frame is a composition of time-frequency domain and sound energy. In the next step, the decomposed frames are segregated into speech-based words. In the last step, the sounds are grouped into a stream form. Before ASA, there were certain circumstances where a chance of errors exist. One of them is an error in the sequential grouping, which results in generating words from two different voices. ASA resolved this error by using sensors that represent individual sounds. The second error is the simultaneous grouping error, which results in sound blending and merging. When the sound-related framework collects sound terms and arranges them in a particular form, concurrent successions of these apparent groupings is called a "sound-related stream" [12]. A stream is often compared to a natural sound that holds on for some time; for example, a man talking, a piano playing, or a puppy barking. However, perceptual mistakes and deceptions are conceivable in extraordinary conditions. One case of this perception error is called spilling or "stream isolation". On the off chance that two sounds, An and B, are quickly exchanged in time, following a couple of moments the discernment, they may appear to "split" when the audience hears two as opposed to one stream of sound. Each stream is compared to the reiterations of one of the two sounds; for instance, an A- can be joined by B-B-B-B- [13]. The propensity towards isolation into streams is supported by the contrast in the acoustical properties of the sounds an A and B. Among the distinctions that support isolation is recurrence (for unadulterated tones), central recurrence (for rich tones), recurrence synthesis, spatial position, and speed of the grouping (quicker successions are isolated promptly). An intuitive site page represents this spilling and the significance of recurrence partition and speed [13]. Computational ASA (CASA) applies the same principle of ASA. Extensive research has been carried out in the development of CASA [14]. In CASA, similar frames are separated via cross-correlations and continuity. Pitch analysis is performed to group the extracted frames [5]. CASA does not support the processing and filtering of low frequencies. Only high frequencies are filtered from the input acoustic signal [15].

**Fig 1:**
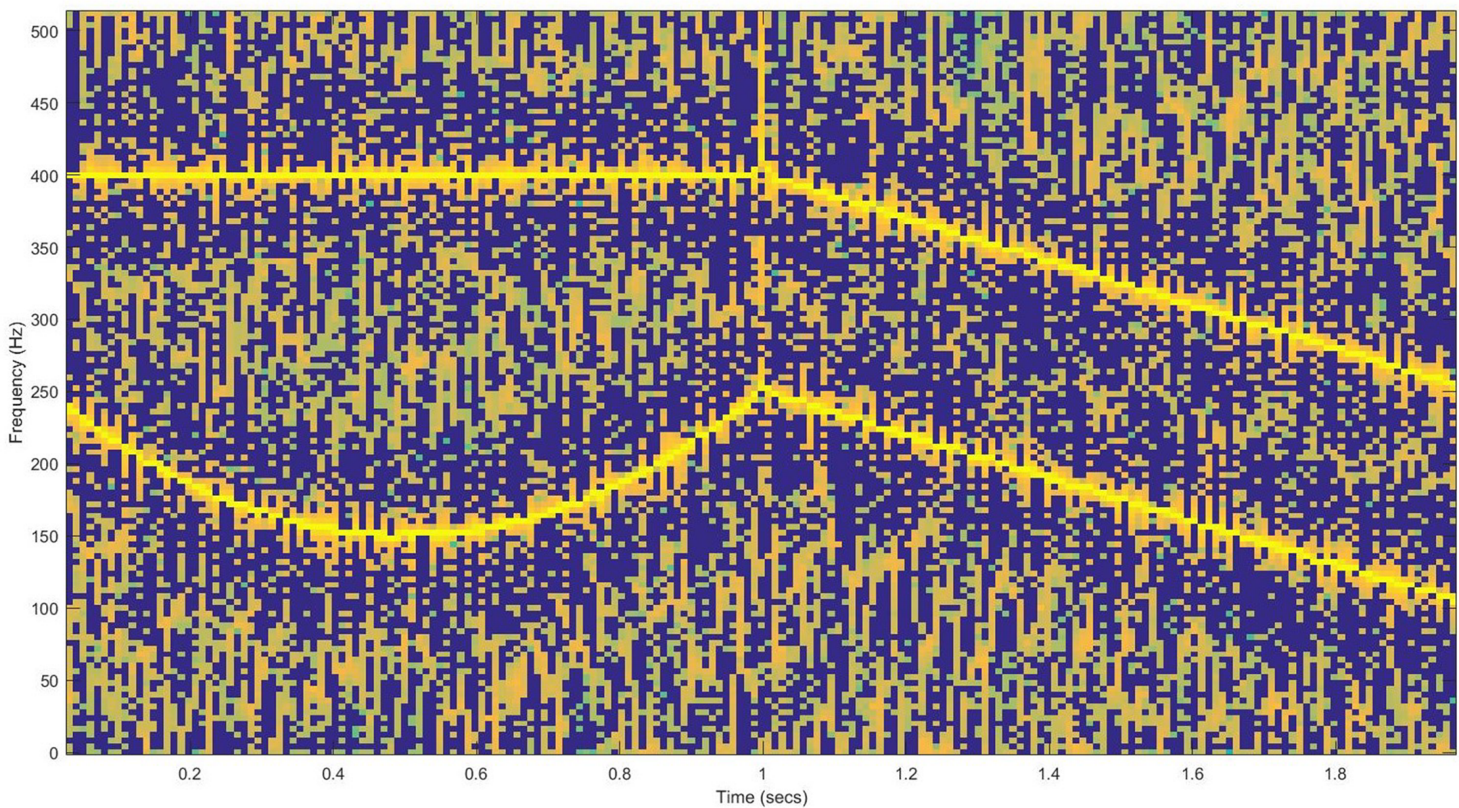
Time-frequency graph of the audio sample.

The MFCC is most widely used in the audio feature field of speech segregation. It is a powerful tool and technique for getting and recognizing specific features of an audio signal. The extracted features of a sample are investigated to identify the unknown audio sample [16]. Computational sound-related scene investigation, which copies the characteristics of the human sound-related framework, can separate target discourse from complex foundation. Henceforth, the CASA approach is a promising approach to manage discourse handling issues under the multi-speaker condition, and its adequacy has been uncovered [3,17]. Chromaprint [18] produced fingerprints and sub-fingerprints of the audio sample, which was down-sampled to 11025 Hz. A short-time Fourier transform (STFT) [18] was applied to the sample with a frame size of 4096, as used by our proposed algorithm. STFT produced a spectrum, which is converted to 12-bins for the classification process [19]. The Echo print [20,21] audio sample was converted to the mono format, and the sample rate was transformed to 11025 Hz. The down-sampled signal was passed through a cosine band filter [9] to produce 128 bands that were grouped into 8 bins of sub-fingerprints, which are then classified. The landmark performs the same steps of the audio sample conversion to a mono format and down-sampling to 11025 Hz. After an STFT is applied, with a window size of 46.4 ms, bin size of 21.5 Hz and a group of 16 bins of sub-fingerprints [1]. Panako [8,21] extracts the local maxima from the audio sample, using the constant Q of the spectrum, and generates the fingerprints. A list of audio identifiers is then generated, updated and maintained for the matching process. Hash values are generated for each fingerprint and sub-fingerprint, which are then used to identify the audio sample. Fig 1 shows a representation of the time-frequency spectrogram of an input audio sample, which contains the sound and music content found in background noise [8].

This research has focused on the removal of stationary noise from an audio sample. Stationary noises are those noises that have the same values at two different time instances, with different events, no matter how far they are [22]. White and pink noises are also stationary noises [22]. White noise has equal energy power per hertz throughout all frequencies, creating a mess of voices in the audio sample [23]. Pink noise is different in all respects from white noise. In pink noise, the power per hertz decreases with increasing frequencies. That is why the frequencies are louder and have more power energy, which decreases the accuracy of speech recognition and segregation applications. To perform speech segregation from the noisy audio mixture, we proposed an algorithm using a layer separation model [24]. Noise is separated from the audio sample using the layered separation process and the DBN classification model to achieve the accuracy of automatic speech segregation [25]. We have removed the noise from the noisy audio sample using hidden layer architecture in which recurrent neural networks (RNN) are employed via hidden layer separation [21]. During the speech segregation, the linguistic content of the audio sample is identified using an MFCC feature algorithm. After the layer separation, we introduced a deep Boltzmann machine technique for classification. An enhanced version of the fisher algorithm is introduced and employed for an efficient classification with improved accuracy [26].

The remaining sections of this paper are organized as follows: Section 2 introduces a critical analysis of the existing state-of-the-art methods for speech segregation. Section 3 presents a comprehensive discussion of the proposed method. Performance evaluation of the proposed method is provided using three standard datasets in Section 4. Finally, Section 5 concludes the paper.

### Proposed methodology

**Fig 2:**
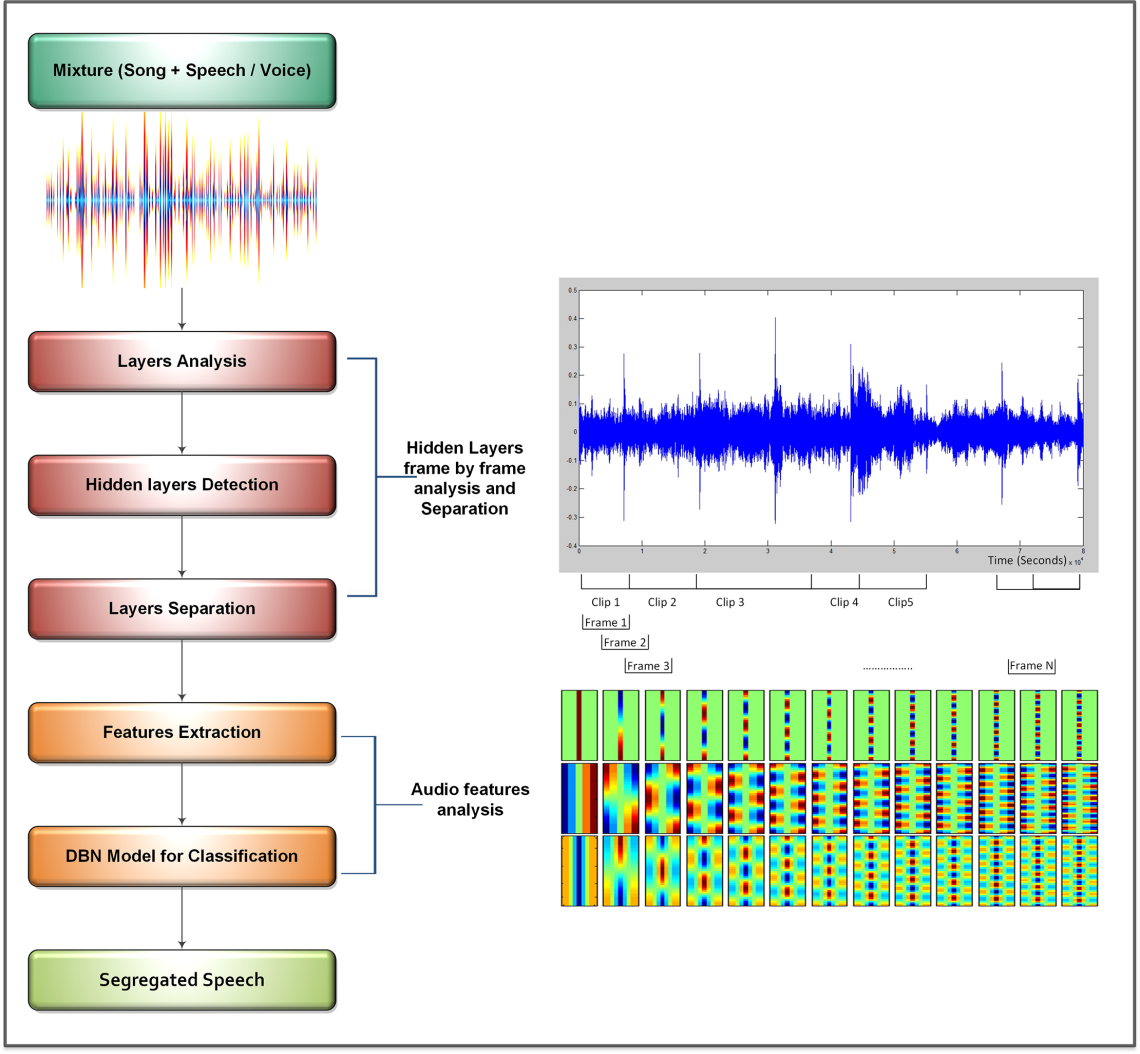
Proposed architecture of speech/music segregation for an audio sample having background noise.

This section provides a comprehensive discussion of the proposed framework. The segregation of speech from an audio signal is a challenging task because of the presence of instrumental or background music and other environmental noise factors. The proposed research work proposes an effective speech segregation method that successfully segregates speech from the input audio signal in the presence of pink and white noise. Fig 2 shows the architectural block diagram of the proposed hybrid model.

**Fig 3:**
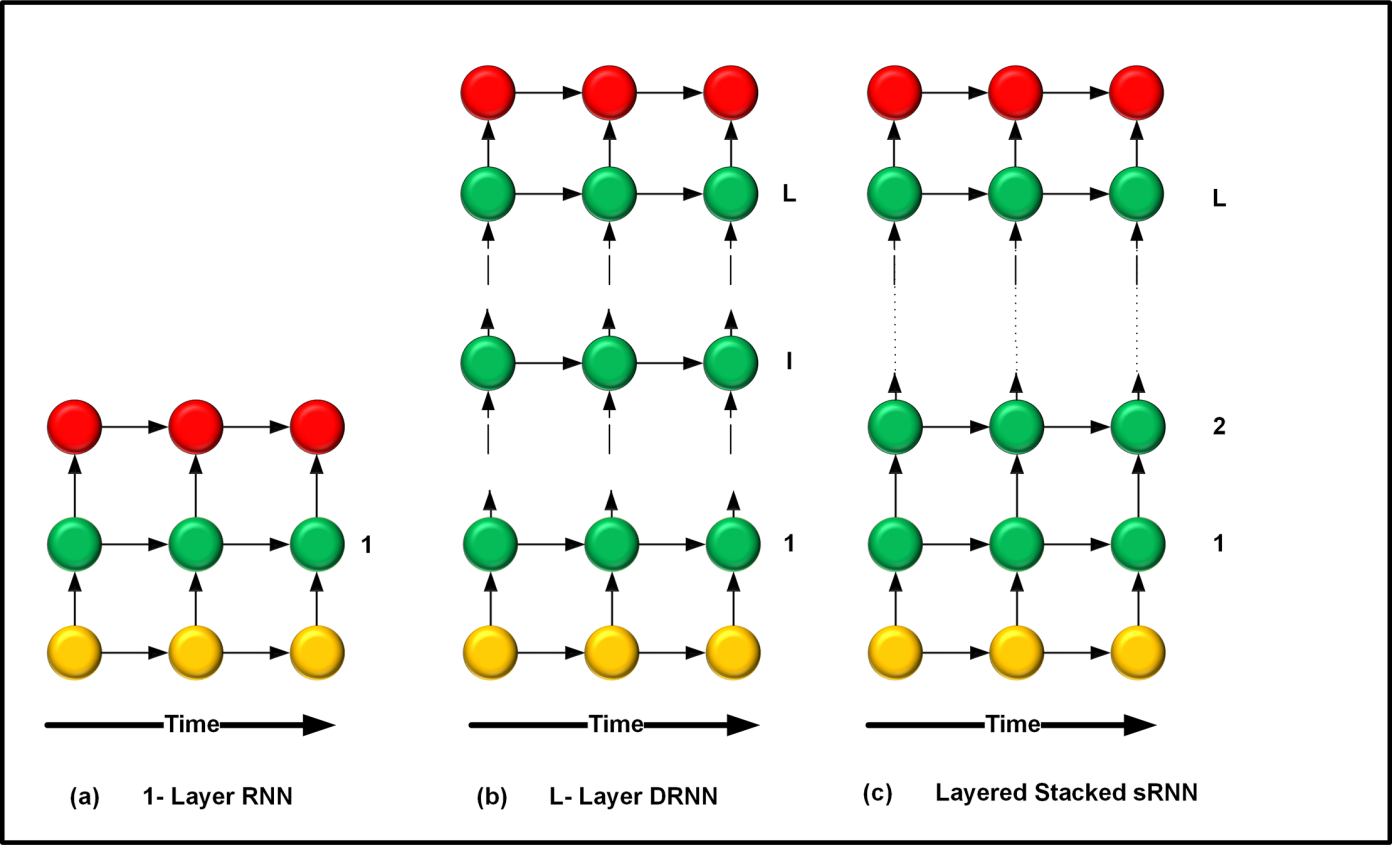
Layer separation architecture.

Fig 2 outlines the different steps of the proposed algorithm. The first step is sample acquisition of the input sound and pre-processing. The second major step is frame-by-frame analysis of the hidden layers and separation of the stationary noise. The last important step is an audio feature extraction; analysis of the extracted features and classification is performed to extract the sound sample and segregate speech. The methodology of the proposed technique is as follows:

1. The first and most important step is to input an audio signal acquisition during the pre-processing. The input audio signal is transformed into a mono sample at a bit rate of 48 kbps. The full-length input audio signal is partitioned into 20-ms sections of the frame that are further processed for segregation.

2. Layered analysis is performed after the transformation of the inputted audio signal. For the audio layer analysis, contextual information is retrieved by applying the recurrent neural network (RNN) model. The layers of analysis are employed to remove the stationary noise successfully. Other techniques that already developed for noise removal failed to remove the noise from the input sample 100% successfully. Thus, for better accuracy and results, layered analysis is introduced in the proposed algorithm.

3. To perform layered analysis and separation, an RNN model is applied. RNN produces layers with temporal contextual information. The layered architecture applied in the proposed method is shown in Fig 3. The arrow represents the connection of metrics [27]. Different coloured circular nodes represent the hidden layers and output frames, as shown in Fig 3. In Fig 3A, the 1–layer RNN implementation is shown, whereas in Fig 3B the DRNN implementation is remembered with L hidden layers and temporal connections. Similarly, Fig 3C shows the stacked recurrent neural network (sRNN) result by considering the full temporal connection.

4. Fig 3 outlines the layer separation model. Circular nodes in each layer represent a hidden layer item. The yellow nodes indicate hidden layer items, green nodes represent the projected required hidden layers, and red nodes illustrate the resultant hidden layer obtained after separation. In an RNN model, stationary audio noise, in the form of hidden layer h^l^, is computed for segregation. In this step, the subsequent frame of signal x is computed after a time interval *t* by using the nonlinear activation function F, as shown in Eq (1).

$$f_{h}=h^{l}x_{t}=F.(w^{l}.h^{l-1}.(x_{t})+b^{l}+u^{l}.h^{l}.(x_{t-1}))$$ (1)

5. In the subsequent frame detection, a hidden layer is extracted. The hidden layer function f_h_ is used in equation (1) where b^l^ is the base layer and used as a reference in the complete segregation process from the digital audio signal matrix x with weight w^l^ at time *t*. u^l^ is an upper layer and is set to zero while computing the first layer. After the initial layer, detection and separation of the whole process is repeated until detection and separation of the *L*^*th*^ layer is used for deep information retrieval using mathematical Eq (2), as shown below. The *L*^*th*^ hidden layer is calculated by using the product of the hidden layer function f_h_ of an audio sample and the signal matrix x_t_ at time t as follows:

$$h_{t}^{l}=f_{h}.(x_{t},h_{t-1}^{l})$$ (2)

where $h_{t-1}^{l}$ is computed by separating Lth hidden layer at time t and the matrix of sample x_t_ from the input noisy audio sample [28].

The output layer is denoted by y_t_ which represents the product of non-linear element wise function ∅ and hyper-tangent $h_{t}^{l}$ of layer L as shown in Eq (3).

$$y_{t}=\emptyset .(h_{t}^{l})$$ (3)

6. The hidden layer ${\hat{y}}_{t}$ is computed as follows:

$${\hat{y}}_{t}=w^{l}{.h}^{l-1}.(x_{t})+c$$ (4)

where y is the predicted hidden layer at time t with constant noise factor c having weighted hidden layer w^l^ of matrix-vector x.

**Fig 4:**
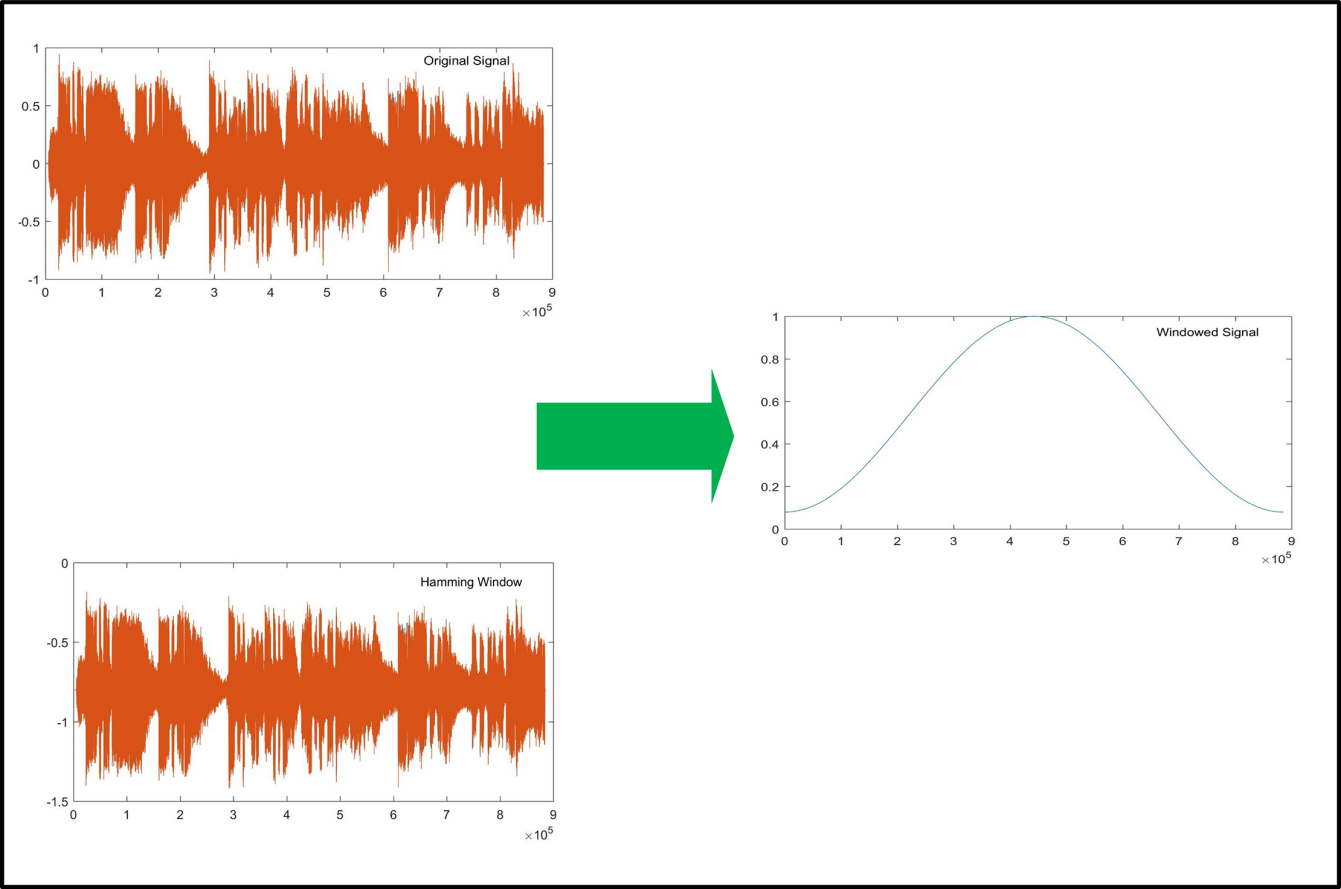
Hamming window of input noisy audio signal.

7. The Mel Filter bank [29] MFCC features are extracted after the layer prediction step. We designed an experiment to compare the performance of the proposed method against existing state-of-the-art methods. For high-quality speech segregation, the Mel-frequency cepstral coefficients (MFCC) features are extracted. MFCC features are commonly employed due to their property of extracting vocal tracts via an envelope, effectively using short-time power spectrum. These vocal tracts identify the speech words from the audio sample. For MFCC, a Hamming window is computed from each frame using an audio sample with N signal points detected from each audio frame. As shown in Fig 4, a hamming window is plotted against time and amplitude.

8. After computing the hamming window, a fast Fourier transform (FFT) is applied to each frame. For a deep audio information and analysis, we have computed 12 coefficients of the Mel scale due to their slow variation of signal characterization of vocal tract shapes and spectrum of shuttered words. The Mel frequency coefficients are a set of features that are used for different pattern recognition tasks that involves human voices. After the transformation, the frequencies of each frame are calculated via

$$Mel(f)=1024*\ln\left(1+\frac{f}{256}\right).$$ (5)

9. The logarithm of the frequency is calculated at a ratio of 256 frames with the product of constant 1024 Hz. For audio feature selection, the critical band energy is determined by converting the linear frequency to a Mel Scale. The Mel Scale is then divided into 20 equally spaced bands. In the critical energy calculation, k-bins of bands using fast Fourier transform are computed as follows:

$$band_{Ampl}=\sum_{k=1}^{n}A_{n}^{2}$$ (6)

10. In the third and final stage, first, a discrete cosine transform (DCT) is applied to each frame spectrum. After the DCT transformation, audio-feature classification is performed. Deep learning models tend to automatically classify more features from big data; however, the dictionary-based classification model is preferred due to its sparse coding functionality. We have used a class-specific sub-dictionary model that classifies speech with up to 90% accuracy. We implemented a sparse dictionary-based learning model where the output was a sparse matrix in the form of its basic elements. These basic elements are called atoms, and a combination of atoms in a single class result in a form of dictionary. We have created a dictionary of bases δ_i_ (words) to learn from the samples. X is a required output sample and is determined by using Eq (7).

$$X\approx \sum_{j=1}^{k}a_{j}\delta_{j}$$ (7)

where δ_j_ is zero for the first item and *a*_*j*_ is constant.

**Fig 5:**
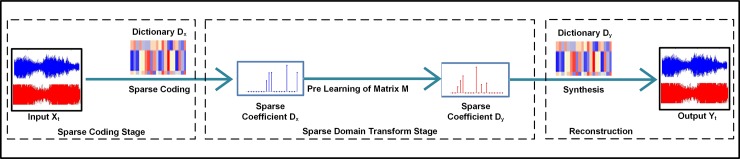
Dictionary-based sparse coding.

11. The δ_j_ term is used to compute the dictionary for music and speech items. A deep belief network is a generative graphical model in machine learning, which is built with multiple layers that are hidden. The Boltzmann machine has many variables representing hidden layers. These variables form a matrix of zeros and ones, but they are mostly zeros. A large dictionary of bases is learned from speech and music samples. There are two ways to train the data by using the dictionary method: the stack method, in which a stack of required layers is created using a deep Boltzmann machine (DBM) technique; or the stack auto-encoder method, which is used for dictionary training. To improve the performance of the classification, we proposed a dictionary-based fisher discrimination algorithm. Fig 5 explains the dictionary-based sparse coding model. Each input signal matrix *X*_*t*_ at time *t* is processed for sparse coding. During the sparse coding step, the signal is matched with a pre-learning matrix *M*. During the processing, a *D*_*x*_ dictionary is used for matching the input matrix *X*_*t*_. S similar match is obtained via the sparse dictionary *D*_*y*_. After a successful match, the resulting output *Y*_*t*_ is used for further processing after reconstruction, as shown in Fig 5.

12. We have employed the learning of classes using structured-based dictionaries rather than a shared dictionary-based model. We supposed the *D*_*i*_ class of dictionary, associated with class *i*, had a total number of *C* classes. With such a dictionary, we could use the reconstruction error for classification [30]. A fisher algorithm is applied for a random permutation of the finite sequence. During permutation, the elements are traversed until all elements present in the group are traversed.

13. The existing fisher algorithm is computationally very expensive and does not offer better accuracy for small databases. To overcome the limitations of the existing fisher algorithm, we have introduced a dictionary-based learning method to the fisher algorithm as presented in Table 1. Existing fisher algorithms use element grouping for classification, which is replaced by a dictionary-learning class model in the proposed algorithm. We trained a number *n* of samples *y* that had a set of training sample classes *A*_*c*_ that is the product of coding coefficient *X* matrix and class *D*. To this end, we propose the following dictionary-based fisher model.

**Table 1:** Dictionary-based fisher algorithm.

Input = Signal Items, Dictionary Items foreach (var D_Dictionary in atoms) { Set Class B = atoms; X = B * D; Update Class B; Update Dictionary items } Repeat Foreach loop until items are closed to dictionary items.

## Results and discussion

This section provides a comprehensive discussion about the results obtained from the proposed method. The accuracy rate and processing time measures are used for performance evaluation. The details of the datasets used to measure the performance of the proposed method are also provided in this section.

**Fig 6:**
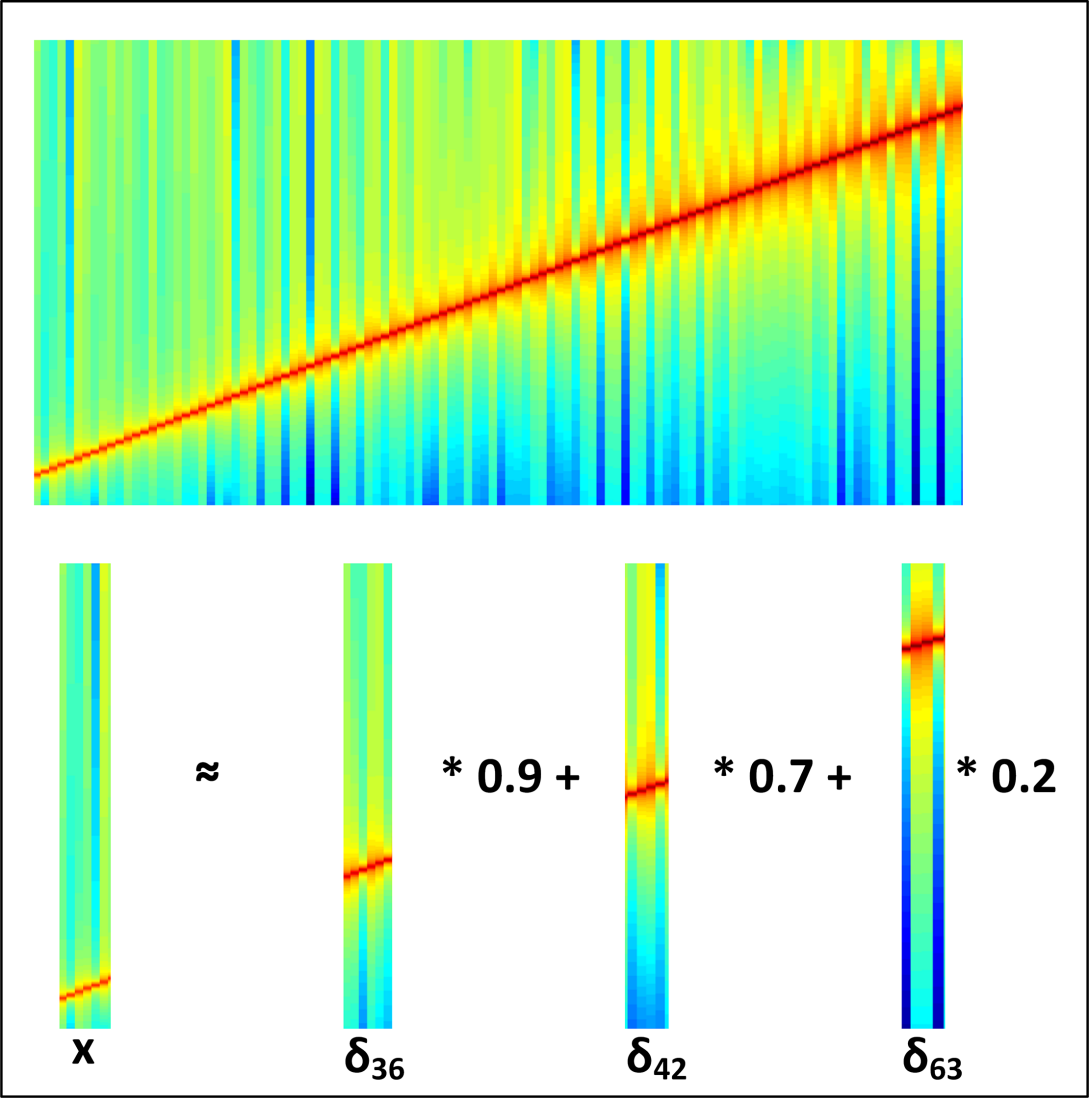
Computation of segments for dictionary item creation.

We used three standard datasets (MIR-1K [31], MusicBrainz [32], and TIMIT [33]) to test the effectiveness of the proposed algorithm in terms of music fingerprinting and speech segregation. The MIR-1K [31] and MusicBrainz [32] datasets are used for songs and musical backgrounds, whereas the TIMIT [33] dataset is used for audio speech mixing. The MusicBrainz dataset contains over 1 million songs in different languages; the MIR-1K [31] contains 25000 songs in Chinese. TIMIT [33] contains the speech data of more than 1630 speakers. In all, 25000 songs from each dataset (MusicBrainz and MIR-1K) and 35000 speech samples were mixed to create a training dataset. For training and testing of the proposed algorithm, we used 16-sec, 12-sec and 8-sec audio samples recorded with a bit rate of 44.1 kHz. For each category, 3160 samples were used for training, and 3350 samples are used for testing. After the segmentation stage, each segment undergoes a matrix X calculation using the summation of delta ∂ for matching. For the computation, bases δ_i_ of different values are multiplied by the matrix of each segment, and then the summation is performed to create the final dictionary item, as shown in Fig 6.

In Fig 6, *x* represents the sum of the product of each frame having designated weight, i.e., 0.9 from one frame, 0.7 from the second frame, and so on, with dictionary basis *δ*_*i*_ for the corresponding frame, as shown above. We designed an experiment to examine the performance of various classifiers trained via MFCC features. The proposed dictionary-based fisher classification model segregates the speech and classifies it with an accuracy of 91.60%, as shown in Table 2. Audio features, including STFT, multi-resolution cochleagram (MRCG) and Chromagram features are evaluated for SVM- and DBN-based proposed models. STFT features produce results with an accuracy of 77.97% for SVM and 81.23% for DBN; MRCG features produce results with an accuracy of 78.6% for SVM and 82.19% for DBN.

**Table 2:** Comparison of accuracy rate of different features extracted with classification algorithms.

Features	Classification algorithm accuracy rate			
	SVM [34]	K-NN [34]	Naive Bayes [34]	DBN (Dictionary-based fisher)
MFCC (Proposed)	88.10%	85.80%	86.20%	91.60%
Bark Scale	82.10%	79.90%	80.10%	87.23%
GFCC	84.10%	81.20%	82.00%	83.20%
MRCG	78.60%	73.20%	76.00%	82.19%
STFT	77.97%	72.12%	71.23%	81.23%
Chromagram	76.78%	71.19%	70.15%	80.67%
Spectral Skewness	75.45%	70.89%	69.67%	79.65%
Spectral Kurtosis	74.32%	69.87%	68.37%	77.37%

We designed an experimental setup to compare the performance of our proposed method against the existing state-of-the-art speech segregation methods. Table 2 shows the results of the comparison of the proposed classification models with existing classification models for acoustic feature extraction. MFCC features are classified with more accuracy while using DBN compared to the existing classification models. Mel Scale (MFCC) features classified the features with a 91.6% accuracy using the DBN model with a bark scale with an 87.3% accuracy. The proposed algorithm is tested on TIMIT, MIR-1K and MusicBrainz datasets.

The accuracy rate and processing time evaluation metrics are used for performance comparison. We trained the Boltzmann machine using unlabelled data and fixed the first layer weights. The results of the unlabelled data are used for training the data. We repeatedly assigned weights until all layers were trained. Table 3 explains the comparison of different algorithms with the methodology used with respect to processing time and accuracy rate. The proposed algorithm used multi-layer separation models with deep neural networks using MFCC features with an accuracy of 91.6% in 1.4 sec processing time. Panako [9] produced results with an accuracy of 87.25% and Echoprint 85.9%, as shown in Table 3.

**Table 3:** Comparison of different speech separation models with respect to methodology used.

Algorithms/ System	Methodology Used	Accuracy Rate (%)	Processing Time (Sec.)
Proposed Model	Multi-layered separation with deep recurrent neural network and MFCC features with DBN model classification	91.60%	1.4
Panako [35]	Local maxima are calculated using constant Q of the spectrogram. Set of hashes is generated for matching	87.25%	2.1
Echoprint [36]	8 bins and sub-fingerprints generated using cosine band filtration	85.9%	2.4
Landmark [30]	16 bins and sub-fingerprints generated using STFT	84.9%	2.6
Chromaprint [21]	12 Hash bins and sub-fingerprints generated using STFT	82.35%	2.7

The computational complexity of the proposed approach is calculated on a laptop with the following specifications: Dell XPS with Intel Pentium (R) Core i7 2.4 GHz microprocessor, integrated GPU, and 8 GB RAM using Windows 10 operating system with 64-bit architecture. The proposed method is implemented in Visual Studio 2017 and SQL Server 2016. Table 4 lists the performance results and the comparison results of tests using the TIMIT and MusicBrainz datasets with respect to short-time objective intelligibility (STOI) and perception evaluation of speech quality (PESQ) for noisy sample inputs and the proposed algorithm. The SNR range is between 3 dB and -3 dB, as shown below. STOI and PESQ increased, as expected, the required output after the proposed algorithm is applied to the noisy input signal. The STOI value varied for a proposed signal between 0.902 and 0.819, whereas the variation for PESQ was between 2.119 and 2.019, as shown in Table 4.

**Table 4:** Performance comparison of TIMIT and MusicBrainz datasets with respect to STOI and PESQ for noisy signal and proposed algorithm.

Dataset	SNR (db.)	Noisy Original Signal		Proposed Algorithm	
		STOI	PESQ	STOI	PESQ
TIMIT	3	0.802	1.395	**0.902**	**2.119**
	0	0.743	1.259	**0.847**	**2.109**
	-3	0.678	1.124	**0.819**	**2.019**
MusicBrainz	3	0.752	1.415	**0.992**	**2.329**
	0	0.857	1.359	**0.917**	**2.207**
	-3	0.669	1.224	**0.899**	**2.289**

Table 5 lists the results of descriptive statistics for a paired sample test. The *T*-value and *P*-value tests are performed using a sample test of the designed experimental setup. Mean standard deviation and standard mean error are computed for a 95% confidence interval difference. *T*-value is 23.05 and *P*-value is 0.000, which shows the significance of the data and the proposed algorithm. We have also performed skewness and kurtosis tests for sample tests, as shown in Table 5. The observed skewness value is -1.031 with a standard error of 0.195, whereas for kurtosis it is -0.950 with standard error of 0.389.

**Table 5:** Descriptive statistics for paired samples test.

Success Ratio for sample test	T—Value	df	P—Value	Mean	Std. Deviation	Skewness		Kurtosis	
				Statistic	Statistic	Statistic	Std. Error	Statistic	Std. Error
	23.050	153	.000	.73	.447	-1.031	.195	-.950	.389

## Conclusion

This paper presents a novel model for speech segregation using a noisy audio sample. While audio speech segregation algorithms are currently used in many applications, speech segregation from an audio signal in the presence of background white and pink noise is a challenging task due to environmental and noisy factors that mislead the contextual information required for audio segregation. This paper proposes an algorithm for speech/music segregation in the presence of background noise. The proposed model represents the combination of a layer model separation method for noise removal and MFCC features for audio contextual information retrieval, which is supported by the DBN model for accurately segregated feature classification. A layered separation approach is applied using recurrent neural network and deep neural network techniques that retrieve contextual information. The separated layers are processed as MFCC features for segregation of the desired audio information. MFCC features resulted in speech segregation with a success rate of up to 91.60% by using the DBN classification model. Deep learning models decrease processing while increasing data size. After removing audio noise and performing speech segregation, applications could be modified to predict the occurrence of speech in the presence of audio noise. This algorithm would be helpful for military-grade applications where audio noise removal is required from audio signals. The proposed work can be extended in terms of deep-learning and speech classification.
